# Keeping obesity status is a risk factor of hypertension onset: evidence from a community-based longitudinal cohort study in North China

**DOI:** 10.3389/fpubh.2023.1170334

**Published:** 2023-04-27

**Authors:** Qiujing Cai, Xiaolei Zhao, Liguang Dong, Xinmin Zhang, Chenglong Wang, Shu Wang, Yi Zhou, Xianliang Zhou, Yanqi Li, Shuyu Wang, Lisheng Liu, Aihua Hu

**Affiliations:** ^1^Center for Non-communicable Disease Management, Beijing Children's Hospital, Capital Medical University, National Center for Children's Health, Beijing, China; ^2^Center for Health Care Management, Peking University Shougang Hospital, Beijing, China; ^3^Beijing Hypertension League, Beijing, China; ^4^Department of Cardiology, Fuwai Hospital, Beijing, China

**Keywords:** obesity status change, hypertension onset, cardiovascular diseases, metabolic disorder, vascular injury and remodeling

## Abstract

**Objective:**

The purpose of our study was to investigate the association of obesity status change with hypertension onset based on a community-based longitudinal cohort study in North China.

**Methods:**

This longitudinal study included 3,581 individuals free of hypertension at baseline in the first survey (2011–2012). All participants were followed up (2018–2019). According to the criteria, a total of 2,618 individuals were collected for analysis. We used adjusted Cox regression models and Kaplan–Meier survival analysis to estimate the association between obesity status change and hypertension onset. Additionally, we applied the forest plot to visualize the subgroup analysis including age, gender, and the differences in some variables between baseline and follow-up. Finally, we conducted a sensitivity analysis to examine the stability of our results.

**Results:**

Over nearly 7 years of follow-up, a total of 811 (31%) developed hypertension. The new hypertension incidence was mostly observed in those who were obese all the time (*P* for trend < 0.01). In the fully adjusted Cox regression model, being obese all the time increased the risk of hypertension by 30.10% [HR 4.01 (95% CI 2.20–7.32)]. The Kaplan–Meier survival analysis revealed the change in obesity status as an important feature to predict the occurrence of hypertension. Sensitivity analysis shows a consistent trend between the change in obesity status and hypertension onset in all populations. Subgroup analysis showed that age above 60 years was an important risk factor for hypertension onset, that men were more likely than women to develop hypertension, and that weight control was beneficial in avoiding future hypertension in women. There were statistically significant differences in ΔBMI, ΔSBP, ΔDBP, and ΔbaPWV between the four groups, and all variables, except baPWV changes, increased the risk of future hypertension.

**Conclusion:**

Our study shows that obese status was notably associated with a significant risk of hypertension onset among the Chinese community-based cohort.

## 1. Introduction

Hypertension has become a major health issue with the increasing aging population and unfavorable healthy behaviors, which contributes to 20% of mortality and 50% of morbidity related to cardiovascular diseases (CVDs) in China (1). Over the past few years, hypertension and obesity have increased worldwide, and hypertension often occurs concurrently with obesity. Moreover, hypertension and obesity are both the major components of metabolic syndrome (MS) that threaten public health, as it interacts with metabolic risk factors to dominate and accelerate the abnormal internal environment homeostasis progression. Despite the increasing global epidemic of obesity, mortality caused by coronary artery disease (CAD) and stroke has declined in the past 10 years, probably as a result of the improved public health management of other CVD risk factors (2). However, the prevalence of hypertension shows a consistently increasing trend among the overweight and obese population (3). The risk of CVD is higher in adults with an elevated body mass index (BMI), but there is little study on whether the obese status change has a relationship with hypertension onset based on the Chinese population since the majority of the earlier research was based on Western population (4).

The rising tendency of overweight and obesity is worrisome and becoming a worldwide challenge. It is widely established that obesity, defined as elevated BMI, has been proven to be associated with a higher risk of hypertension (5). Moreover, because obesity is occurring at increasingly younger ages, it is likely to translate into a high cumulative incidence of hypertension (6). Currently, individuals are experiencing a large cumulative exposure to excess adiposity obtained in their lifetime, thus, it is very important to understand the effect of obesity status change on hypertension onset. This raises the question of whether the obesity status change is more detrimental to future hypertension risk. A more in-depth perception of the relationship between obesity and hypertension would be critical to the better management of abdominal obesity-related cardiometabolic risk, thus offering an additional chance for the primary prevention of CVD (7).

Emerging evidence has demonstrated that obese individuals were at higher risk of hypertension later in life (8), though no information about how obesity status changes affect hypertension onset was provided in those studies. Hence, our study aimed to study the association between obesity status change and hypertension onset based on a community-based longitudinal cohort in North China. In this research, we analyzed longitudinal data to determine if there was an association between obesity status changes with the consequent risk of hypertension onset.

## 2. Methods

### 2.1. Study population and design

This longitudinal cohort study of the Shougang community in Beijing in North China is a prospective cohort, which was designed to assess the determinants and progression of CVD between 2011 and 2019 among 2,618 participants aged from 18 to 98 years. We recruited participants from the employee and retiree population of the community. Beginning from the study's inception, participants conducted healthy assessments and questionnaires for demographic features, lifestyle factors, anthropometric measurements, and blood tests. The baseline was conducted from 2011 to 2012, and the enrolled individuals were re-examined from 2018 to 2019. All participants were provided written informed consent for inclusion in this cohort.

**Figure 1:**
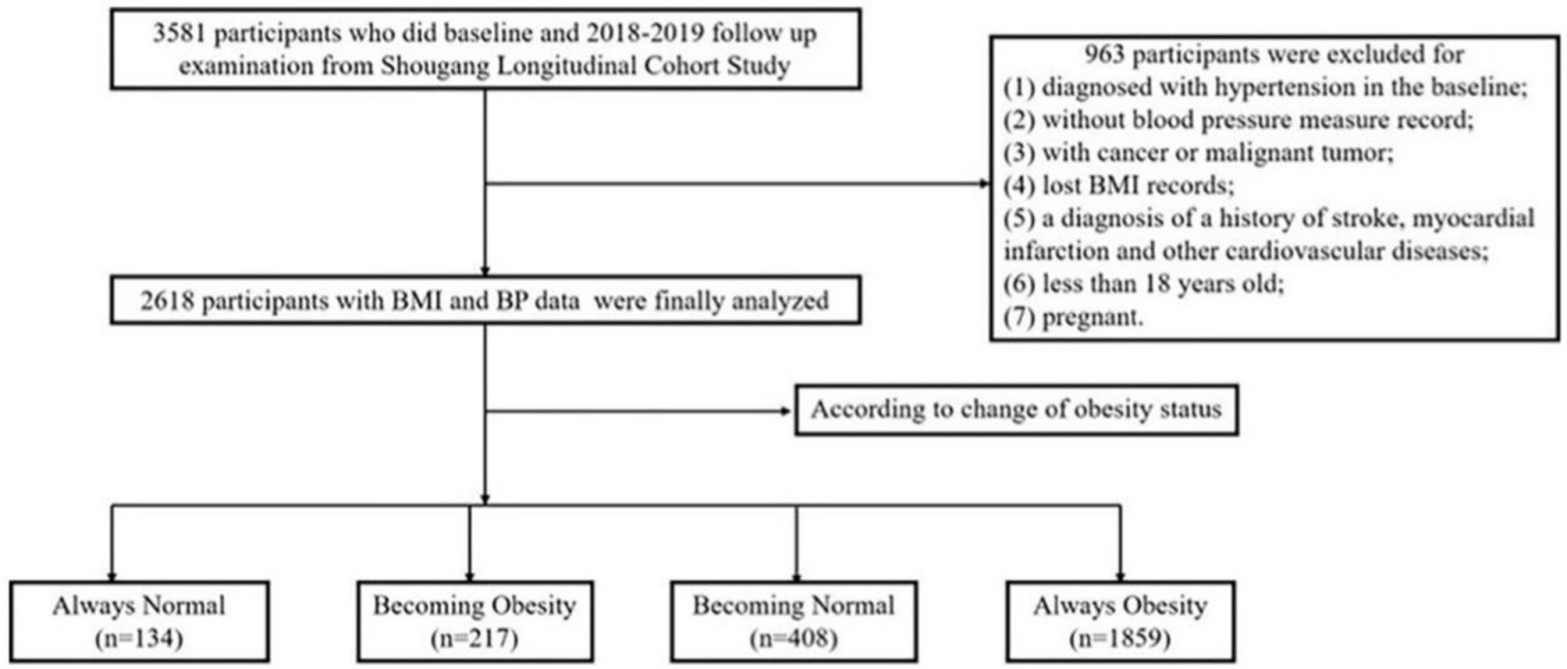
Flow chart of this cohort study.

Figure 1 illustrates the flow chart for screening eligible participants in 3581 subjects. The subjects who had complete follow-up records were included in our study. The exclusion criteria were as follows: (1) participants diagnosed with hypertension in baseline; (2) without blood pressure measure records; (3) with cancer or malignant tumor; (4) lost BMI records; (5) diagnosis of a history of stroke, myocardial infarction, or other CVD; (6) less than 18 years old; and (7) pregnant.

Finally, a total of 2,618 participants who had normal blood pressure were enrolled in our study. The survey protocols, instruments, and the process for obtaining informed consent were approved by the Ethics Committee of Beijing Hypertension League Institute (Ethical Approval No. 2017-102), and each participant provided written informed consent. Our study conformed with the principles of the Declaration of Helsinki and the following checklist in accordance with STROBE Statement 2019 (Supplementary Table 4).

### 2.2. Definition of obesity status change

The change in obesity status means that according to the BMI change from baseline to 2018, we divided the population into four groups: Group 1 (always having a normal weight, which means from baseline to follow-up, this group's weight kept in the normal range), Group 2 (from normal weight to becoming obesity, which means this group had normal weight at first, but being fat finally), Group 3 (from obesity to become normal weight, which means this group was obese at first, and then becoming normal), and Group 4 (keeping obesity all the time, which means this group had been obese during 7 years). Those four groups represented four obesity statuses in our study.

Body mass index (BMI) was calculated as weight (kg) divided by height squared (m^2^) and subsequently categorized as low (<18.5 kg/m^2^), normal (18.5–23.9 kg/m^2^); overweight (24.0–27.9 kg/m^2^), or obese (≥28 kg/m^2^) according to the criteria of the expert consensus on obesity prevention and treatment in China (9). In a personal assessment, weight (to the nearest 0.1 kg) and height (to the nearest 0.1 cm) were determined by standardized equipment, and BMI was then calculated as described above.

### 2.3. Definition of hypertension onset

According to multiple previous studies (10–12), we defined hypertension onset as follows. After the baseline examination without hypertension, individuals who were diagnosed with hypertension in follow-up examination were reported hypertension onset.

Hypertension criteria (13) included: (1) average systolic blood pressure (SBP) ≥140 mmHg or diastolic blood pressure (DBP) ≥ 90 mmHg; (2) self-reported hypertension; and (3) current administration of antihypertensive drugs within 2 weeks.

Systolic and diastolic blood pressures were measured three times by one trained examiner using an automatic blood pressure monitor (Omron HEM-7200 Monitor) in the right arm according to a standardized protocol. The average blood pressure was calculated accordingly.

### 2.4. Assessment of covariates

We used a validated questionnaire to obtain participants' information, including demographic information, metabolic biomarkers, and health behavioral factors to control the related bias. For smoking and drinking, we combined data on current and previous smoking and drinking status. Body height and weight were measured using a standard method by calibrated apparatus. Brachial-ankle pulse wave velocity (baPWV) was examined by the Omron non-invasive vascular screening device (BP-203RPEIII). Demographic information (age and gender) and health behaviors (smoking status and alcohol consumption) were accessed from face-to-face household interviews using structured questionnaires. After household interviews, venous blood was collected by trained staff. The blood samples were transported to the Peking University Shougang Hospital in Beijing and stored at −80°C refrigerator. Metabolic biomarkers were acquired following a standard protocol of biochemical detection, including triglycerides (TGs), low-density lipoprotein (LDL), high-density lipoprotein (HDL), and total cholesterol (TC).

### 2.5. Statistical analysis

The statistical analysis were performed according to the recommendation of the American Heart Association Scientific Publication Committee (14). The missing covariates were filled with mean to reduce selection bias. Normally distributed variables (decided by the Kolmogorov–Smirnov test) are presented as mean ± standard deviation (SD). Categorical variables were provided as frequencies with percentages. The baseline characteristics were compared among the obesity status change groups quartiles by one-way ANOVA test (normal distribution), and the difference of hypertension onset based on four groups was analyzed by chi-square test (categorical variables).

A multivariable Cox regression analysis was conducted to estimate hazard ratios (HRs) and 95% confidence intervals (95% CI) for the association between obesity status change and hypertension onset. First, we adjusted for age and gender, and then in the minimally adjusted model, we adjusted age, sex, income, smoking status, alcohol status, outdoor activity status, and amount of adding salt in food. In the final Cox models, we adjusted for the following potential confounders: age, sex, income, smoking status, alcohol status, outdoor activity status, amount of adding salt in food, family history of hypertension, and family history of high cholesterol. Additionally, stratified analyses were performed to evaluate the possible effect modification of subgroups according to age, gender, and the change in BMI, SBP, DBP, and baPWV. Furthermore, a sensitivity analysis based on the exclusion of participants who had a family history of hypertension was performed to test the robustness of the findings. We conducted the *P* for interaction analysis as well to examine the sex-to-obesity status interaction in association with hypertension onset. Finally, we illustrated the outcomes of the Kaplan–Meier survival analysis to dig into the influence of obesity status change and hypertension onset. A two-tailed *P* < 0.05 was considered to be statistically significant in all analyses. All data analyses were performed using SPSS 26.0 (SPSS Inc., Chicago, Illinois, United States), and all figures were performed by R software (version 4.2.1), GraphPad Prism 8, and Origin 2021.

## 3. Results

### 3.1. Clinical characteristics of the study population

**Figure 2:**
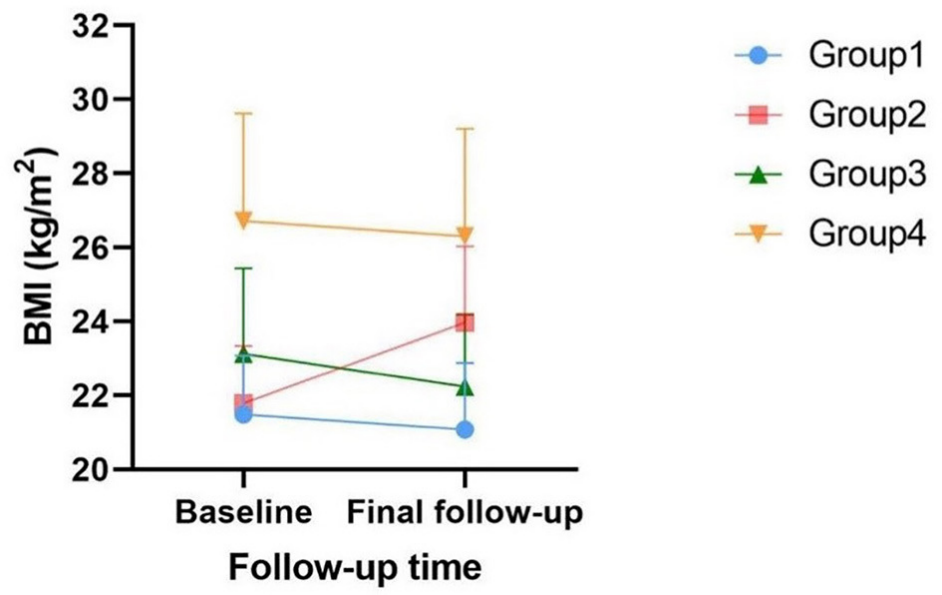
Visualization of four groups defined by BMI changes.

The clinical characteristics of the study population are presented in Table 1 according to obesity status change. This cohort study consisted of 2,618 participants with a mean age of 55.57 years, and 32.3% of them were male participants. Four groups defined by BMI changes from baseline to the final follow-up are shown in Figure 2. In the baseline survey, although all individuals were without hypertension, the other three groups' SBP and DBP elevated when compared with Group 1's SBP and DBP (118.11 ± 10.70, 68.73 ± 7.38). Consistently, Group 2 (120.51 ± 11.28, 69.79 ± 7.93), Group 3 (121.93 ± 10.82, 70.86 ± 7.82), and Group 4 (125.33 ± 8.82, 72.83 ± 7.48) were also increasing by groups. Interestingly, Group 3 was the population that lost weight successfully, and their blood pressure was higher than Group 2 in which the participants were fatter than baseline. This interesting finding might illustrated that once people became obese, their blood pressure would increase. Moreover, compared with Group 1 and Group 2, individuals from Group 3 who were obese at baseline had superior TC, LDL, TG, waist–hip ratio (WHR), baPWV, and decreased HDL. The characteristics of Group 4 who were keeping obesity status were always at the supreme level.

**Table 1:** Baseline characteristics of the total participants according to different BMI change groups.

**Characteristics**		**Total**	**Group 1**	**Group 2**	**Group 3**	**Group 4**
Population	*n* (%)	2,618	134 (5.10%)	217 (8.30%)	408 (15.60%)	1,859 (71.00%)
Age	Mean ± SD	55.57 ± 7.37	52.08 ± 6.79	54.60 ± 8.06	55.48 ± 7.05	55.96 ± 7.32
Sex					
Male	*n* (%)	845 (32.30%)	3 (2.20%)	37 (17.10%)	69 (16.90%)	736 (39.60%)
Female	*n* (%)	1,773 (67.70%)	131 (97.80%)	180 (82.90%)	339 (83.10%)	1,123 (60.40%)
Diagnosis BMI	Mean ± SD	25.49 ± 3.32	21.48 ± 1.59	21.78 ± 1.55	23.13 ± 2.30	26.72 ± 2.91
WHR	Mean ± SD	0.89 ± 0.06	0.80 ± 0.03	0.81 ± 0.03	0.88 ± 0.04	0.91 ± 0.06
Smoking status					
Never	*n* (%)	2,224 (84.95%)	131 (97.76%)	204 (94.01%)	373 (91.42%)	1,515 (81.50%)
Yes	*n* (%)	394 (15.05%)	2 (1.49%)	13 (5.99%)	35 (8.58%)	344 (18.50%)
Blood pressure					
SBP	Mean ± SD	124.04 ± 9.72	118.11 ± 10.70	120.51 ± 11.28	121.93 ± 10.82	125.33 ± 8.82
DBP	Mean ± SD	72.06 ± 7.67	68.73 ± 7.38	69.79 ± 7.93	70.86 ± 7.82	72.83 ± 7.48
baPWV	Mean ± SD	1,513.72 ± 269.86	1,406.37 ± 244.91	1,465.39 ± 274.64	1,482.95 ± 252.69	1,533.85 ± 271.67
TC	Mean ± SD	5.32 ± 1.58	5.38 ± 1.04	5.15 ± 1.01	5.37 ± 0.92	5.32 ± 1.77
TG	Mean ± SD	1.51 ± 1.22	1.07 ± 0.51	1.11 ± 0.99	1.40 ± 1.36	1.61 ± 1.23
HDL	Mean ± SD	1.48 ± 0.48	1.81 ± 0.38	1.71 ± 0.41	1.60 ± 0.37	1.40 ± 0.49
LDL	Mean ± SD	3.21 ± 0.82	3.14 ± 0.86	2.98 ± 0.80	3.24 ± 0.77	3.24 ± 0.82

BMI, body mass index; SBP, systolic blood pressure; DBP, diastolic blood pressure; baPWV, brachial-ankle pulse wave velocity; TC, total cholesterol; TG, triglyceride; HDL, high-density lipoprotein; and LDL, low-density lipoprotein.

### 3.2. The crude prevalence of hypertension onset

**Figure 3:**
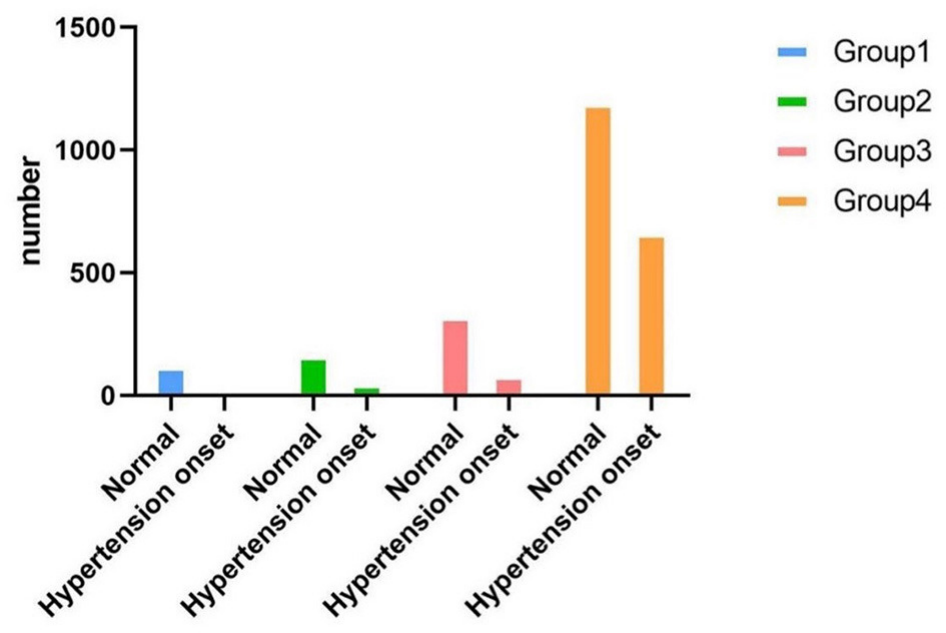
Bar plot shows the crude prevalence of hypertension onset situation in four groups.

During a 7-year follow-up, 811 participants (31%) developed hypertension. Table 2, Figure 3 summarize the hypertension onset situation of four groups. The hypertension onset was more frequently observed in Group 4 (*n* = 665/*N* = 1,859, 35.80%) where the participants were keeping obesity status all the time. The hypertension onset was seen in Group 2 (*n* = 51/*N* = 217, 23.50%) higher than in Group 3 (*n* = 84/*N* = 408, 20.60%). The hypertension onset was seen lowest in Group 1 (*n* = 11/*N* = 134, 8.20%). The chi-square test was adopted with a statistically significant difference (χ^2^=78.74, *P* < 0.01). We found that keeping obese had a strong association with hypertension onset, and becoming fat was observed to have a higher risk for future hypertension than keeping a normal weight and becoming thin.

**Table 2:** Crude prevalence of hypertension onset in four groups.

**Groups**		**Total**	**Group 1**	**Group 2**	**Group 3**	**Group 4**	***χ^2^***	***P-*value**
Normal	*n* (%)	1,807 (69.00%)	123 (91.80%)	166 (76.50%)	324 (79.40%)	1,194 (64.20%)	78.74	< 0.01
Hypertension onset	*n* (%)	811 (31.00%)	11 (8.20%)	51 (23.50%)	84 (20.60%)	665 (35.80%)		

Group 1: always normal weight; Group 2: becoming obesity; Group 3: becoming normal weight; and Group 4: always obesity.

### 3.3. Risk of obesity status change and hypertension onset

**Figure 4:**
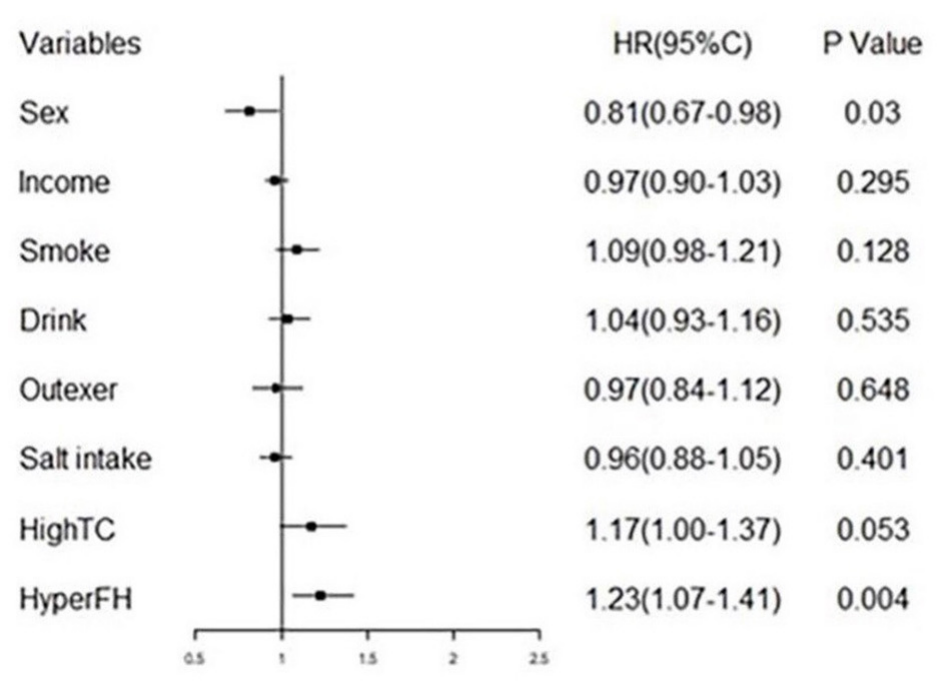
Forest plot of fully adjusted variables in COX regression analysis model (model 3) shows risk of different obesity status and hypertension.

**Figure 5:**
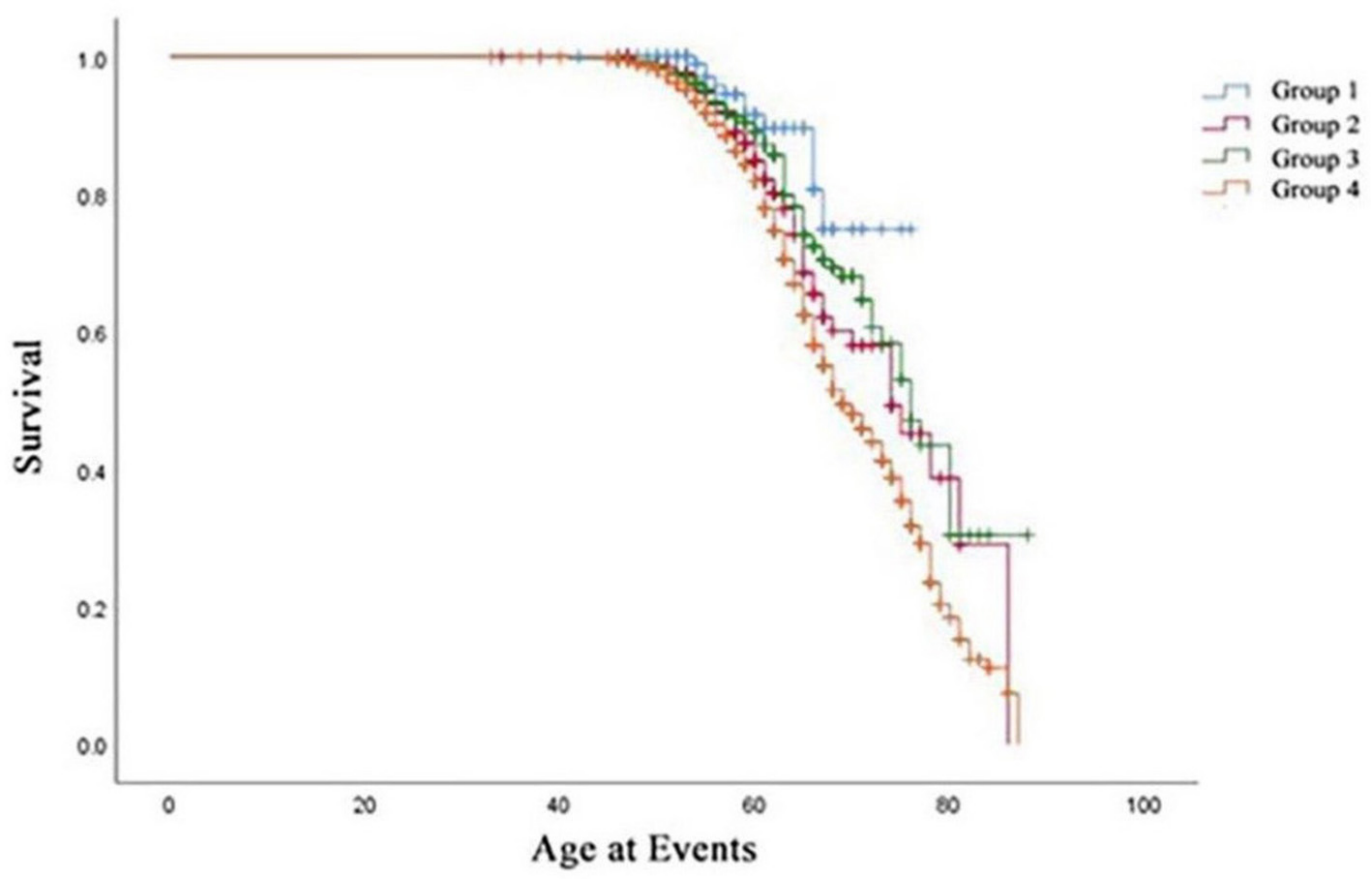
Kaplan-Meier estimates of survival in the four groups.

Table 3 summarizes the Cox regression analysis of the association between obesity status change and hypertension onset. The other groups had an increased risk of developing hypertension in comparison to Group 1. In the fully adjusted model, participants in Group 2 [HR 2.82 (95%CI 1.47–5.42); *P* < 0.01] exhibited a 2.82-fold risk, Group 3 [HR 2.29 (95%CI 1.22–4.31); *P* < 0.05] exhibited a 2.29-fold risk, and Group 4 [HR 4.01 (95%CI 2.20–7.32); *P* < 0.01] exhibited a 4.01-fold risk of developing hypertension when setting the Group 1 as reference. Moreover, the risk factors involved in model 3 (fully adjusted model) were shown by a forest plot (Figure 4). The results of the Kaplan–Meier survival analysis showing in Figure 5 revealed that the odds of cumulative risk of hypertension onset increased by years in participants from Groups 1 to Group 4, and the percentage of increase was not equal among groups. By calculating the cumulative risk probability of developing hypertension onset, the results showed that four groups were statistically significant compared to the other groups.

**Table 3:** Cox models indicate the association of obesity status changes with hypertension onset.

	**Hypertension onset**					
	**Model 1**		**Model 2**		**Model 3**	
	**HR (95% CI)**	***P-*** **value**	**HR (95% CI)**	***P-*** **value**	**HR (95% CI)**	***P-*** **value**
Group 1	1		1		1	
Group 2	2.79 (1.45, 5.46)	<0.01	2.80 (1.46, 5.39)	<0.01	2.82 (1.47, 5.42)	<0.01
Group 3	2.30 (1.23, 4.32)	<0.05	2.29 (1.22, 4.30)	<0.05	2.29 (1.22, 4.31)	<0.05
Group 4	4.05 (2.22, 7.38)	<0.01	4.05 (2.22, 7.38)	<0.01	4.01 (2.20, 7.32)	<0.01
Age	0.57 (0.55, 0.58)	<0.01	0.57 (0.55, 0.60)	<0.01	0.57 (0.55, 0.60)	<0.01
Sex, female	0.79 (0.69, 0.91)	<0.01	0.85 (0.71, 1.03)	0.09	0.81 (0.67, 0.98)	<0.05
Income	…	…	0.97 (0.91, 1.04)	0.36	0.97 (0.90, 1.03)	0.30
Smoking status	…	…	1.09 (0.98, 1.22)	0.10	1.09 (0.98, 1.21)	0.13
Alcohol status	…	…	1.04 (0.93, 1.17)	0.50	1.04 (0.93, 1.16)	0.54
Outdoor activity	…	…	0.98 (0.85, 1.13)	0.82	0.97 (0.84, 1.12)	0.65
Salt intake	…	…	0.95 (0.87, 1.05)	0.32	0.96 (0.88, 1.05)	0.41
Family history of hypertension	…	…	…	…	1.23 (1.07, 1.41)	<0.01
Family history of high cholesterol	…	…	…	…	1.17 (0.99, 1.37)	0.05

Model 1 (crude model) adjusted for age and sex.

Model 2 (minimally adjusted model) adjusted for age, sex, income, smoking status, alcohol status, outdoor activity, and salt intake.

Model 3 (fully adjusted model) fully adjusted, with the addition of adjustments for family history of hypertension and family history of high cholesterol.

### 3.4. Subgroups analysis

#### 3.4.1. Age subgroup analysis

**Figure 6:**
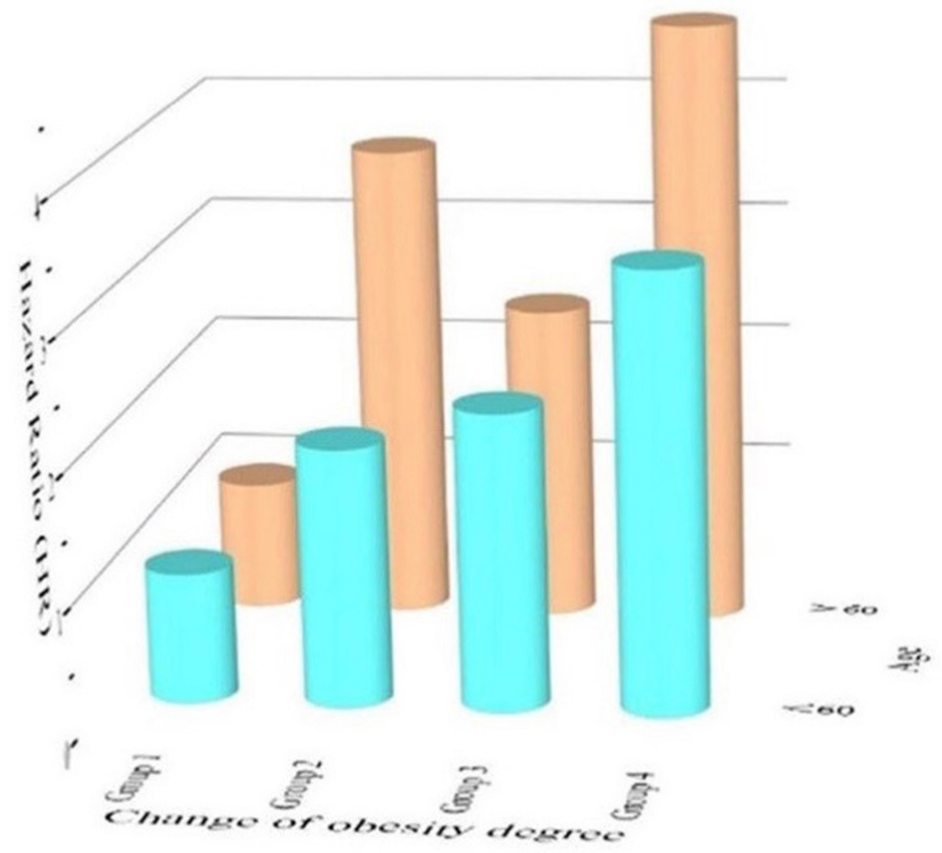
3D bar plot showing the cox regression analysis of age under 60 years old and age over 60 years old.

**Figure 7:**
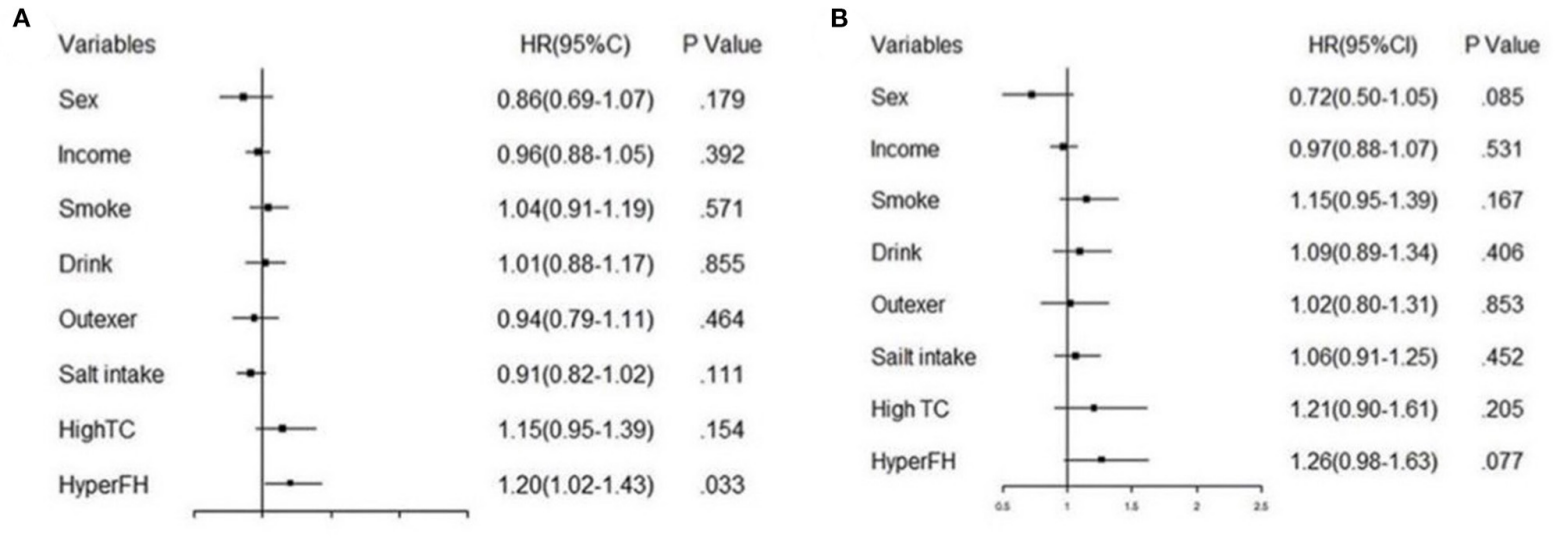
The forest plot of fully adjusted Cox model analysis of age subgroups. **(A)** shows the result of subgroup under 60 years old and **(B)** illustrates the result of subgroup over 60 years old.

As age was a significant variable in the Cox regression model, we divided the population into two subgroups based on age (<60 years and ≥60 years). Figure 6 shows that age over 60 years was a significant risk factor contributing to future hypertension, especially can be observed that the HR of age over 60 years was high in Group 2 (HR 3.69) and Group 4 (HR 4.67). Moreover, other variables analyzed above were shown in the next two forest plots with HRs and 95%CI (Figures 7A, B). Figure 7A shows the result of age under 60 years, and Figure 7B shows the result of age over 60 years.

#### 3.4.2. Sex subgroup analysis

**Figure 8:**
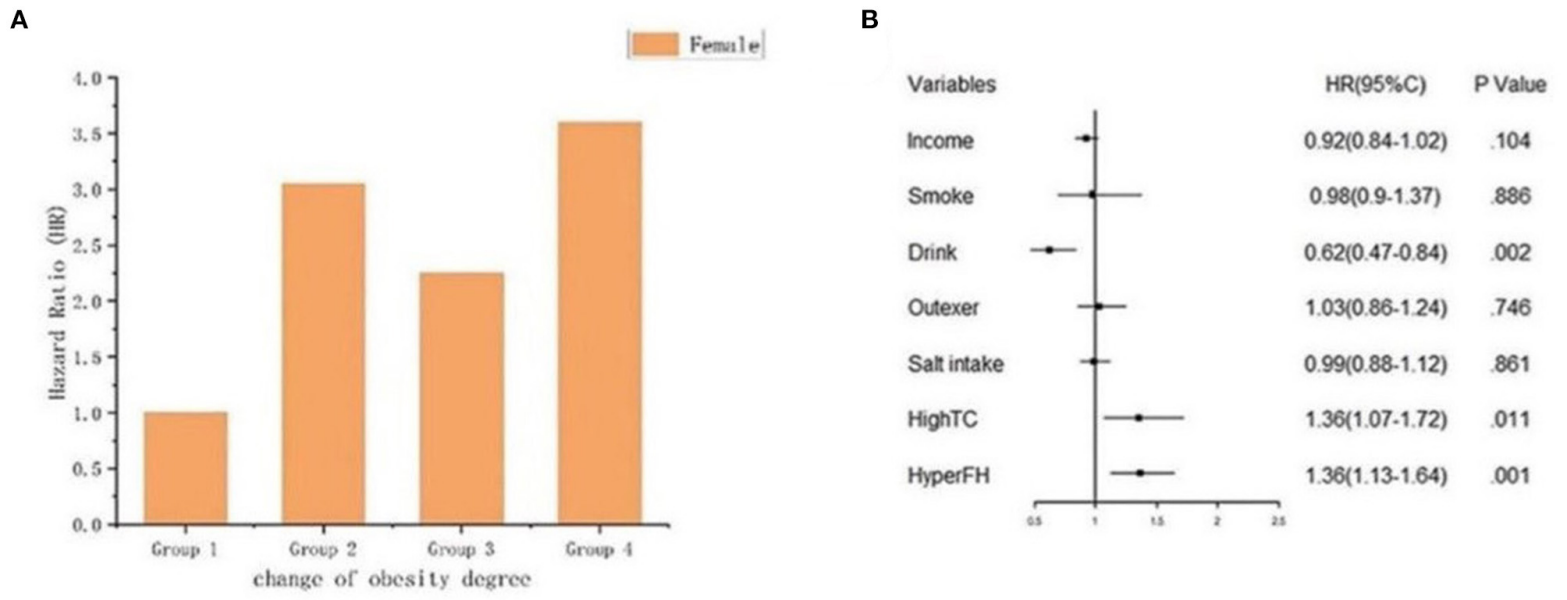
The graph indicated the risk of female with hypertension onset. **(A)** Bar plot; **(B)** Forest plot of the association between female and hypertension onset based on cox regression analysis. The association was adjusted for income, smoke, drink, outdoor activity, salt intake, high total cholesterol history and hypertension family history.

Because sex was a significant variable in the Cox regression model, female participants had a lower risk of developing future hypertension [HR 0.81 (95%CI 0.67–0.98)] than male participants. Therefore, we analyzed the risk of the female participants to have hypertension onset (Figures 8A, B), Figure 8A shows the HR of each group, Group 2 [HR 3.05 (95%CI 1.57–5.92)], Group 3 [HR 2.25 (95%CI 1.19–4.27)], and Group 4 [HR 3.60 (95%CI 1.97–6.58)] respectively. Figure 8B shows the association between female and hypertension onset based on Cox regression analysis. The outcomes displayed that when the female gender was taken into account, the risk of the decreased BMI (Group 3) with the hypertension onset was attenuated. Moreover, remaining obese (Group 4) and becoming obese (Group 2) in female participants might increase the risk of future hypertension. We also classified the participants into two subgroups according to gender based on the joint associations of sex and obesity status change with hypertension onset (*P* for interaction = 0.009) (Supplementary Table 1).

### 3.5. Association of the ΔBMI, ΔSBP, ΔDBP, and ΔbaPWV between baseline to final follow-up with hypertension onset within four groups

Table 4 showed the ΔBMI, Δ SBP, ΔDBP, and ΔbaPWV between four groups had significant statistical differences (*P* < 0.01); however, after fully adjusting by age, sex, income, smoking, drinking, outdoor activity, salt intake, high cholesterol, and family history of hypertension, the HR (95%CI), 1.06 (1.02, 1.10), 1.05 (1.05, 1.06), 1.06 (1.06, 1.07), and 1.00 (1.00, 1.00), respectively, which indicated that there were no statistical significant that baPWV change made to hypertension onset. The variables adjusted contributed to the risk of future hypertension are shown in Supplementary material.

**Table 4:** Association of the ΔBMI, ΔSBP, ΔDBP, and ΔbaPWV between baseline and follow-up with hypertension onset according to different obesity statuses.

**Characteristics**		**Total**	**Group 1**	**Group 2**	**Group 3**	**Group 4**
BMI 2012 (kg/m^2^)	Mean ± SD	25.49 ± 3.32	21.48 ± 1.59	21.78 ± 1.55	23.13 ± 2.30	26.72 ± 2.91
BMI 2018 (kg/m^2^)	Mean ± SD	25.04 ± 3.33	21.07 ± 1.80	23.97 ± 2.07	22.24 ± 1.95	26.30 ± 2.91
ΔBMI (kg/m^2^)	Mean ± SD	−0.44 ± 1.73	−0.45 ± 1.21^**^	0.23 ± 1.57^**^	−0.89 ± 1.81^**^	−0.42 ± 1.73^**^
**BP 2012**						
SBP	Mean ± SD	124.04 ± 9.72	118.11 ± 10.70	120.51 ± 11.28	121.93 ± 10.82	125.33 ± 8.82
DBP	Mean ± SD	72.06 ± 7.67	68.73 ± 7.38	69.79 ± 7.93	70.86 ± 7.82	72.83 ± 7.48
**BP 2018**						
SBP	Mean ± SD	128.30 ± 15.04	118.27 ± 14.32	124.36 ± 14.20	124.44 ± 15.34	130.32 ± 14.58
DBP	Mean ± SD	77.95 ± 9.03	73.68 ± 8.16	75.63 ± 8.79	75.59 ± 8.82	79.04 ± 8.93
Δ**BP**						
ΔSBP	Mean ± SD	4.23 ± 13.15	0.15 ± 10.72**	3.91 ± 11.85^**^	2.46 ± 12.58^**^	4.94 ± 13.49^**^
ΔDBP	Mean ± SD	5.87 ± 7.99	4.94 ± 6.51^**^	5.86 ± 7.79^**^	4.76 ± 7.27^**^	6.18 ± 8.23^**^
baPWV 2012	Mean ± SD	1,513.72 ± 269.86	1,406.37 ± 244.91	1,465.39 ± 274.64	1,482.95 ± 252.69	1,533.85 ± 271.67
baPWV 2018	Mean ± SD	1, 619.48 ± 308.96	1,487.16 ± 259.25	1,582.41 ± 316.82	1,580.22 ± 296.94	1,641.97 ± 310.47
ΔbaPWV	Mean ± SD	105.76 ± 224.82	80.79 ± 184.45^**^	117.01 ± 197.99^**^	97.27 ± 200.29^**^	108.12 ± 235.22^**^

P-value < 0.05 is statistically significant.

* P < 0.05,

** P < 0.01, and

*** P < 0.001.

## 4. Discussion

Hypertension is an independent risk factor for cardiovascular events, and it is also the major modifiable risk factor for CVD and premature mortality. Current estimates suggest that there are 435.3 million people with prehypertension worldwide (15), accounting for 41.3% of the population aged over 18 years. Studies on hypertension have primarily focused on weight status during childhood and adulthood (16). However, to the best of our knowledge, there is little study on the association of obesity status change with hypertension onset among the adult population in the Chinese community.

In our community-based, longitudinal cohort study, we found that 811 participants were newly diagnosed with hypertension after nearly 7 years of follow-up. Notably, the most important outcome of our study is that maintaining obesity status (Group 4) was associated with the highest HR of future hypertension, whereas losing weight to become normal weight (Group 3) was associated with a slightly higher risk of hypertension onset than status from normal weight to obesity (Group 2). Our finding is in accordance with recent studies (17), indicating that keeping obesity status is an important risk factor for hypertension onset. A possible explanation for this might be that patients keeping obesity status are accompanied by sympathetic nerve excitation (18), which results in the amount of norepinephrine, angiotensin II, and adrenaline being released in their body causing vasoconstriction so that their blood pressure is rising over time. Previous studies (19, 20) have shown that there are many other internal mechanisms of obesity that affect the hypertension onset, such as renin–angiotensin–aldosterone system (RAAS) activation, water and sodium retention, vascular endothelial contraction, and even dysfunction through mediating oxidative stress and abnormal inflammatory response. Whereas, taking the “obesity paradox” into account (21), which is that individuals with mildly higher BMI are associated with better survival and fewer cardiovascular events, our study's finding is in contrast to it. We thought obesity has a bad risk effect on hypertension onset. The reasons for differences between the contrast results might be the following aspects, such as limited studies on the relationship between obesity and hypertension onset, the ambiguous elucidated pathophysiological process of obesity-induced hypertension, and the co-pathogenesis under it. Hence, more case–control studies and cohort studies on more people are needed to evaluate the correlation between obesity and hypertension onset later.

Additionally, the risk of hypertension onset was continuously attenuated with each group according to female gender and age under 60 years. Interestingly, our results were opposite to Li's research (8), in which they observed that participants older than 60 were negatively associated with an increased incidence of hypertension. The various results between other studies and ours might be caused by the different study population composition. Our study had nearly 67.7% female participants, and they had 32.1% female participants involved in their study. The huge gender difference made our research outcomes contrary to their research. Nonetheless, these current findings are likely to be prone to bias caused by self-reporting or thinking of body weight, or they were limited to a finite age range that did not extend beyond middle age. In addition, our study indicated a gender difference in hypertension onset with different obesity status changes. In line with the previous report, Tara's study has shown that male participants were more likely than female participants to develop a variety of obesity-related diseases, such as diabetes, hypertension, and other CVD (22). The underlying pathogenic mechanism might be that the inflammation level in male participants is higher than in female participants (23), which causes the function of endothelial cells to decrease without any responses to vascular or immune stimulation. However, in female participants, the vascular endothelial cells show the sustainable ability to respond to the changing inflammation level even though keeping high-fat diet stress. Meanwhile, this evidence may expound the possible mechanism behind the influence of obesity on hypertension onset.

The most interesting founding of our research is that the population who has an obesity history, no matter how they lost weight by any means, their risk to develop future hypertension was higher than the population who was always keeping a normal weight, especially higher than the population who were normal at first with obesity status later. It indicated no matter developing into obesity status or from obesity to normal weight, as long as obesity had occurred in one's lifespan, blood pressure would be abnormal. Studies have found that this is caused by the hidden danger of immune cell abnormalities during the obesity period (24). We speculate that this might be due to a large amount of free fatty acids in the circulatory system of obese individuals, and these fatty acids can directly change the innate immune cells including monocytes and macrophages into inflammatory phenotypes, which are retained in the aging process of mice through “epigenetic memory” (25). These patterned macrophages then travel in the body, where they initiate inflammatory programs that promote age-related hypertension. One study analyzed adipose tissue from obese, lean, and normal mice, and the researchers found that after a while on a high-fat diet (HFD), adipose-related macrophages (ARMs) in adipose tissue were observed to develop toward a pro-inflammatory phenotype, with many inflammatory genes becoming significantly expressed in these cells (26), including tumor necrosis factor-α (TNF-a), interleukin-1b (IL-1b), and interleukin-6 (IL-6). The two studies above provide a deep insight into the mechanism of obesity incidence's effect on system metabolic change. In other words, adipose tissue macrophages produce cytokines due to a history of obesity and maintain a pro-inflammatory state after weight loss. Those studies may explain the reason why people who losing weight after being obesity still have high risks to deliver future hypertension.

Moreover, decreased physical activity, obesity genes, intrauterine epigenetics, environmental toxins, and high-fat and fructose diets lead to insulin resistance, obesity, hypertension, and vascular dysfunction, suggesting a vicious cycle throughout the lifespan. A complex interaction of endocrine factors (27), cytokines, vascular cellular components (28), extracellular matrix (28), perivascular adipose tissue (29), and immune cells (30) could be seen within the progression of hypertension. Diet-induced obesity creates conditions for impaired endothelial nitric oxide synthase activation (31), vascular cell-specific mineralocorticoid and increased aldosterone plasma level, and decreased nitric oxide bioavailability (32) leading to increased vascular permeability and inflammation, leukocyte adhesion, increased vascular constriction, tissue remodeling, and fibrosis (6). Those may indicate the communication behind obesity and hypertension. Diet-induced obesity in early life will trigger the continuous reprogramming of the innate immune system (33), which will persist long after the normalization of metabolic abnormalities and have a lasting impact on individual health status. Therefore, individuals who are not obese need to try their best to avoid obesity and reduce the possibility of leaving epigenetic abnormal cells.

Above all, obesity and hypertension are major global public health problems and disease burdens. Obesity has been recognized as an important risk factor for hypertension. Our study shows the association of obesity status change with hypertension onset. First, we found that keeping obesity status was the most important risk among the four statuses for delivering hypertension onset. Second, female gender and age under 60 years were two protective factors for different obesity status groups to have future hypertension, and losing weight was more significant in the female population. Last but not least, what is interesting is that once obesity happens in one's life, the blood pressure level could not recover to a normal state easily.

## 5. Limitation

There are several potential limitations to our study. The major limitation of our study is that we only used BMI as the standard to define obesity status change. BMI-defined obesity has heterogeneous to some extent because BMI could not reflect regional body fat distribution. Nevertheless, BMI is still the most frequently used metric for identifying obesity, which had a minimal influence on our results. Admittedly, the present study is also limited by the lack of variables that influence hypertension onset possibly in the cohort database (12, 34), such as heart rate, estimated glomerular filtration rate (eGFR), and fasting blood glucose (FBG). Further studies with those variables and multi standards defined obesity status change are still warranted.

## 6. Conclusion

In the present study, we observed a trend toward an association between obesity status change and hypertension onset. Keeping obese status was associated with the highest risk of hypertension onset. Losing weight after being obese was associated with a higher risk of hypertension onset than being at normal weight at first and then becoming fat. Moreover, keeping a normal weight is always the best choice to be away from future hypertension. Awareness of weight control and prevention from becoming obese may contribute to hypertension risk management in clinical practice.

## Data availability statement

The original contributions presented in the study are included in the article/Supplementary material, further inquiries can be directed to the corresponding author.

## Ethics statement

Written informed consent was obtained from the individual(s) for the publication of any potentially identifiable images or data included in this article.

## Author contributions

QJC and XLZ designed the whole study and modified the manuscript. XMZ, CLW, SW, and YQL collected and tidied up the clinical information with data analysis. YZ, XLZ, LGD, SYW, LSL, and AHH revised the manuscript and gave professional advice. QJC wrote the manuscript. All authors reviewed this manuscript.

## References

[1] Zhang M Shi Y Zhou B Huang Z Zhao Z Li C. Prevalence, awareness, treatment, and control of hypertension in China, 2004-18: findings from six rounds of a national survey. BMJ. (2023) 380:e071952. 10.1136/bmj-2022-071952 36631148 PMC10498511

[2] GBD 2017 Causes of Death Collaborators. Global, regional, and national age-sex-specific mortality for 282 causes of death in 195 countries and territories, 1980-2017: a systematic analysis for the Global Burden of Disease Study 2017. Lancet. (2018) 392:1736–88. 10.1016/S0140-6736(18)32203-7 30496103 PMC6227606

[3] Mills KT Stefanescu A He J. The global epidemiology of hypertension. Nat Rev Nephrol. (2020) 16:223–37. 10.1038/s41581-019-0244-2 32024986 PMC7998524

[4] Laurent S Chatellier G Azizi M Calvet D Choukroun G Danchin N. SPARTE study: normalization of arterial stiffness and cardiovascular events in patients with hypertension at medium to very high risk. Hypertension. (2021) 78:983–95. 10.1161/HYPERTENSIONAHA.121.17579 34455813 PMC8415523

[5] El Meouchy P Wahoud MA-O Allam SA-O Chedid RA-O Karam W Karam S. Hypertension related to obesity: pathogenesis, characteristics, and factors for control. Int J Mol Sci. (2022) 23:12305. 10.3390/ijms232012305 36293177 PMC9604511

[6] Cote AT Harris KC Panagiotopoulos C Sandor GG Devlin AM. Childhood obesity and cardiovascular dysfunction. J Am Coll Cardiol. (2013) 62:1309–19. 10.1016/j.jacc.2013.07.042 23954339

[7] Arnett DK Blumenthal RS Albert MA Buroker AB Goldberger ZD Hahn EJ. ACC/AHA guideline on the primary prevention of cardiovascular disease: executive summary: a report of the american college of cardiology/American Heart Association task force on clinical practice guidelines. Circulation. (2019) 140:e563–e95. 10.1161/CIR.0000000000000724 30879339 PMC8351755

[8] Li W Fang W Huang Z Wang X Cai Z Chen G. Association between age at onset of overweight and risk of hypertension across adulthood. Heart. (2022) 108:683–8. 10.1136/heartjnl-2021-320278 35190372 PMC8995813

[9] Wang Y. Expert consensus on obesity prevention and treatment in China. Chin Prev Med. (2022) 23:321–39. 10.16506/j.1009-6639.2022.05.001 35589563

[10] Niiranen TJ McCabe EL Larson MG Henglin M Lakdawala NK Vasan RS. Heritability and risks associated with early onset hypertension: a multigenerational, prospective analysis in the Framingham Heart Study. BMJ. (2017) 357:j1949. 10.1136/bmj.j1949 28500036 PMC5430541

[11] Niiranen TJ Henglin M Claggett B Muggeo VMR McCabe E Jain M. Trajectories of blood pressure elevation preceding hypertension onset: an analysis of the framingham heart study original cohort. JAMA Cardiol. (2018) 3:427–31. 10.1001/jamacardio.2018.0250 29562081 PMC5875333

[12] Wang C Yuan Y Zheng M Pan A Wang M Zhao M. Association of age of onset of hypertension with cardiovascular diseases and mortality. J Am Coll Cardiol. (2020) 75:2921–30. 10.1016/j.jacc.2020.04.038 32527401

[13] Williams B Mancia G Spiering W Agabiti Rosei E Azizi M Burnier M. 2018 ESC/ESH Guidelines for the management of arterial hypertension. Eur Heart J. (2018) 39:3021–104. 10.1097/HJH.0000000000001940 30165516

[14] Althouse AD Below JE Claggett BL Cox NJ de Lemos JA Deo RC. Recommendations for statistical reporting in cardiovascular medicine: a special report from the American Heart Association. Circulation. (2021) 144:e70–91. 10.1161/CIRCULATIONAHA.121.055393 34032474 PMC12850682

[15] Egan BM Stevens-Fabry S. Prehypertension–prevalence, health risks, and management strategies. Nat Rev Cardiol. (2015) 12:289–300. 10.1038/nrcardio.2015.17 25687779

[16] Zhao M Bovet P Xi B. Weight status change from adolescence to young adulthood and the risk of hypertension and diabetes mellitus. Hypertension. (2020) 76:583–8. 10.1161/HYPERTENSIONAHA.120.14882 32594799

[17] Thompson P Logan I Tomson C Sheerin N Ellam T. Obesity, sex, race, and early onset hypertension: implications for a refined investigation strategy. Hypertension. (2020) 76:859–65. 10.1161/HYPERTENSIONAHA.120.15557 32755414

[18] Carnagarin R Gregory C Azzam O Hillis GS Schultz C Watts GF. The role of sympatho-inhibition in combination treatment of obesity-related hypertension. Curr Hypertens Rep. (2017) 19:99. 10.1007/s11906-017-0795-1 29080925

[19] Schütten MT Houben AJ de Leeuw PW Stehouwer CD. The link between adipose tissue renin-angiotensin-aldosterone system signaling and obesity-associated hypertension. Physiology. (2017) 32:197–209. 10.1152/physiol.00037.2016 28404736

[20] Matsuda M Shimomura I. Increased oxidative stress in obesity: implications for metabolic syndrome, diabetes, hypertension, dyslipidemia, atherosclerosis, and cancer. Obes Res Clin Pract. (2013) 7:e330–41. 10.1016/j.orcp.2013.05.004 24455761

[21] Lavie CJ Milani RV Ventura HO. Obesity and cardiovascular disease: risk factor, paradox, and impact of weight loss. J Am Coll Cardiol. (2009) 53:1925–32. 10.1016/j.jacc.2008.12.068 19460605

[22] Rudnicki M Pislaru A Rezvan O Rullman E Fawzy A Nwadozi E. Transcriptomic profiling reveals sex-specific molecular signatures of adipose endothelial cells under obesogenic conditions. iScience. (2023) 26:105811. 10.1016/j.isci.2022.105811 36624843 PMC9823135

[23] Stanhewicz AE Wenner MM Stachenfeld NS. Sex differences in endothelial function important to vascular health and overall cardiovascular disease risk across the lifespan. Am J Physiol Heart Circul Physiol. (2018) 315:H1569–h88. 10.1152/ajpheart.00396.2018 30216121 PMC6734083

[24] Hata M Andriessen E Hata M Diaz-Marin R Fournier F Crespo-Garcia S. Past history of obesity triggers persistent epigenetic changes in innate immunity and exacerbates neuroinflammation. Science. (2023) 379:45–62. 10.1126/science.abj8894 36603072

[25] Bekkering S Saner C Riksen NP Netea MG Sabin MA Saffery R. Trained immunity: linking obesity and cardiovascular disease across the life-course? Trends Endocrinol Metab. (2020) 31:378–89. 10.1016/j.tem.2020.01.008 32305098

[26] Mangum KD Gallagher KA. Obesity confers macrophage memory. Science. (2023) 379:28–9. 10.1126/science.adf6582 36603093

[27] Kraemer WJ Ratamess NA Hymer WC Nindl BC Fragala MS. Growth hormone(s), testosterone, insulin-like growth factors, and cortisol: roles and integration for cellular development and growth with exercise. Front Endocrinol. (2020) 11:33. 10.3389/fendo.2020.00033 32158429 PMC7052063

[28] Cai Z Gong Z Li Z Li L Kong W. Vascular extracellular matrix remodeling and hypertension. Antioxid Redox Signal. (2021) 34:765–83. 10.1089/ars.2020.8110 32460598

[29] Oriowo MA. Perivascular adipose tissue, vascular reactivity and hypertension. Medical principles and practice: international journal of the Kuwait University. Health Sci Centre. (2015) 24 (Suppl. 1): 29–37. 10.1159/000356380 24503717 PMC6489082

[30] Agita A Alsagaff MT. Inflammation, immunity, and hypertension. Acta Med Indones. (2017) 49:158–65. 28790231

[31] Zhou H Gao F Yang X Lin T Li Z Wang Q. Endothelial BACE1 impairs cerebral small vessels via tight junctions and eNOS. Circ Res. (2022) 130:1321–41. 10.1161/CIRCRESAHA.121.320183 35382554

[32] Leo F Suvorava T Heuser SK Li J LoBue A Barbarino F. Red blood cell and endothelial eNOS independently regulate circulating nitric oxide metabolites and blood pressure. Circulation. (2021) 144:870–89. 10.1161/CIRCULATIONAHA.120.049606 34229449 PMC8529898

[33] Mischke MA-O Arora T Tims S Engels E Sommer N van Limpt K. Specific synbiotics in early life protect against diet-induced obesity in adult mice. Diabetes Obes Metab. (2018) 20:1408–18. 10.1111/dom.13240 29460474 PMC5969090

[34] Aladin AI Al Rifai M Rasool SH Keteyian SJ Brawner CA Michos ED. The association of resting heart rate and incident hypertension: the henry ford hospital exercise testing (FIT) project. Am J Hypertens. (2016) 29:251–7. 10.1093/ajh/hpv095 26112864

